# A Hybrid-Dimensional Iterative Coupled Modeling of Lubrication Flow in Deformable Geological Media with Discrete Fracture Networks

**DOI:** 10.3390/ma19071444

**Published:** 2026-04-04

**Authors:** Yue Xu, Tao You, Qizhi Zhu

**Affiliations:** 1 Key Laboratory of Ministry of Education for Geomechanics and Embankment Engineering, Hohai University, Nanjing 210098, China; xuyue_hhu@hhu.edu.cn; 2 College of Civil and Transportation Engineering, Hohai University, Nanjing 210098, China; 3 Department Geoenergy, Montanuniversität Leoben, 8700 Leoben, Austria

**Keywords:** non-conforming discretization, lower-dimensional crack, hybrid-dimensional coupling strategy, fracture width estimation

## Abstract

Fluid-driven fracture processes are central to the development of subsurface energy systems such as geothermal and hydrocarbon reservoirs. Although phase-field formulations have become a widely used tool for describing fracture initiation and growth, the diffuse representation of cracks makes it difficult to resolve flow behavior accurately inside discrete fracture networks (DFNs) and to represent hydro-mechanical coupling in a sharp-interface sense. This study develops a hybrid-dimensional iterative framework for lubrication-flow simulation in deformable fractured geomaterials. By leveraging phase-field point clouds together with non-conforming discretization schemes for both the solid matrix and fracture domains, the proposed framework enables the dynamic reconstruction of evolving fracture networks. The theoretical formulation and numerical implementation of the coupling strategy are presented in detail. Hydraulic benchmark examples verify the performance of the fluid flow solver under various physical conditions. The classical Sneddon problem and Khristianovic–Geertsma–de Klerk (KGD) model are employed to validate the solid deformation solver, confirming accurate predictions of crack opening displacement and mesh independence in fracture width calculation. Additional simulations with complex pre-existing fracture patterns further demonstrate the applicability of the framework to coupled hydro-mechanical analysis in fractured media.

## 1. Introduction

Fracture propagation driven by pressurized fluid strongly influences the performance of subsurface energy systems, including shale, petroleum, and geothermal reservoirs [1,2]. For this reason, the numerical simulation of coupled flow-deformation behavior in fractured rocks has become an important approach for investigating fracture growth, fluid migration, and network evolution under different geological settings [3].

Among various computational approaches, phase-field models have attracted considerable attention due to the ability to capture complex fracture nucleation, branching, and coalescence phenomena without the explicit tracking of crack surfaces [4,5,6]. These methods provide a diffuse approximation of the fracture, allowing robust numerical implementation in continuous solid media. Recent studies have further extended phase-field hydraulic fracture modeling to porous media, including formulations for fracture nucleation and propagation, hydro-mechanical coupling, and interactions with pre-existing natural fractures [7,8,9]. However, the diffuse nature brings a significant challenge to accurately resolve fluid flow within discrete fracture networks, where localized aperture and permeability are critical to describing lubrication flow [10]. Moreover, the smeared representation of fractures complicates the modeling of pre-existing natural fracture topology, which governs flow paths in geological reservoirs [11,12]. Owing to the implicit representation of discrete fractures in smeared damage formulations, most phase-field approaches approximate fracture effects by enhancing permeability within damaged regions, while omitting the explicit solution of fluid flow on lower-dimensional DFNs; see the review article [13]. In parallel, recent developments in fractured porous media flow have shown that hybrid-dimensional formulations provide an efficient way to represent fracture–matrix flow exchange while preserving the lower-dimensional nature of discrete fractures [14,15]. Santillán et al. [16] have taken an important step by solving the flow problem on a sharp crack path, but that formulation is not straightforward for complex fracture networks. Zhao et al. [17] have proposed a hydro-mechanical strategy, in which discrete fractures are regularized within a phase-field setting, although the required local mesh refinement near the fracture region may substantially increase computational expense.

To overcome these limitations, a natural alternative is to represent fractures as lower-dimensional manifolds embedded in the surrounding rock matrix, i.e., lines in two dimensions and surfaces in three dimensions [18,19]. To accurately resolve the matrix-fracture interface, the fracture discretization must be sufficiently refined and compatible with the adjacent matrix grid [20,21,22]. This requirement necessitates the use of locally refined and unstructured meshes to match the explicit geometry of the fractures [23]. However, such mesh refinement leads to several drawbacks, including high computational cost, reduced flexibility and meshing complexity. The hybrid models enable a detailed characterization of the fracture aperture and hydraulic property, and allow for the direct solution of lubrication or Darcy–Stokes flow equations along the fracture domain. However, coupling such DFN representations with the mechanical deformation of the surrounding solid media remains challenging, especially when non-conforming discretization is employed to handle evolving fracture geometries [24,25]. In particular, recent hydro-mechanical studies have emphasized the numerical difficulty of consistently coupling flow, deformation, and fracture evolution under embedded or non-conforming discretizations [26,27,28]. The iterative schemes capable of exchanging information between the matrix and fractures are therefore essential to ensure consistency in hydro-mechanical coupling and stability in numerical implementation.

In the present study, we introduce a hybrid-dimensional iterative strategy for lubrication flow in deformable media with discrete fracture networks. This framework integrates non-conforming discretization for fractures and reservoirs, enabling independent and flexible mesh optimization. The iterative coupling procedure ensures the consistent transfer of hydraulic pressure and mechanical displacement across the matrix–fracture interface, satisfying equilibrium and mass conservation conditions during the iterative coupling process. The remainder of the paper is organized as follows. Section 2 details the mathematical formulation of the proposed hybrid-dimensional hydro-mechanical system for phase-field fracture. Section 3 describes the numerical procedure of the iterative strategy, as well as employing the fixed-stress split scheme to stabilize the flow equation. Section 4 validates the hydraulic solver and mechanical solver against benchmark cases, illustrating precise crack opening displacement as well as mesh independence in fracture width calculation. Moreover, this section discusses application in discrete fracture networks and demonstrates the model’s adaptability for tackling coupled hydro-mechanical processes. Section 5 provides a brief discussion of the proposed hybrid method, with regard to the current limitations and possible directions for future research. The final Section 6 closes the paper with concluding remarks.

## 2. Methods

### 2.1. Balance Laws

**Figure 1:**
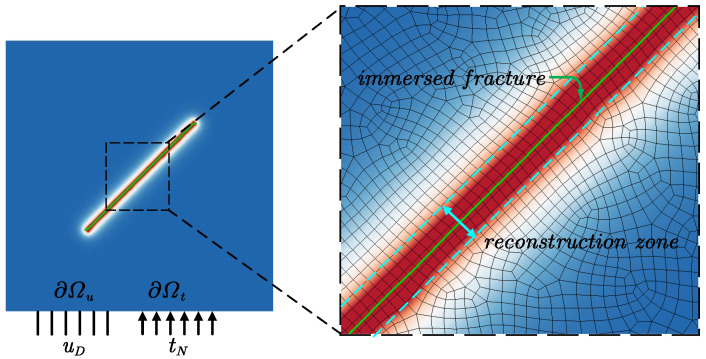
Schematic diagram of phase-field model and the immersed fracture.

As illustrated in the left part of Figure 1, consider a cracked brittle solid $\Omega \subset R^{n}(n\in \left\{2,3\right\})$ with boundary ∂Ω. $u_{D}$ and $t_{N}$ denote the Dirichlet and Neumann boundary conditions, respectively, defined on $\partial \Omega_{u}$ and $\partial \Omega_{t}$, which together form a complete partition of the boundary, i.e., $\partial \Omega_{u}\cup \partial \Omega_{t}=\partial \Omega$ and $\partial \Omega_{u}\cap \partial \Omega_{t}=⌀$. Neglecting body forces, the linear momentum balance law reads as Equation (1):

(1) ∇·σ=0 in Ω∖Γ,

where $\sigma :=C_{e}:\nabla^{s}u$, with $\nabla^{s}u$ denoting the linearized strain, defined as

$$\nabla^{s}u:=\frac{\nabla u+{\left(\nabla u\right)}^{T}}{2}.$$

The boundary conditions and continuity of stress at the fracture surface yield

(2) $$\begin{array}{c} \left\{\begin{array}{c} u=u_{D}\;\mathrm{on}\;\partial \Omega_{u}\\ \sigma \cdot n_{t}=t_{N}\;\mathrm{on}\;\partial \Omega_{t}\\ \sigma^{\pm }\cdot n_{\Gamma^{\pm }}=-p_{f}n_{\Gamma^{\pm }}\;\mathrm{on}\;\Gamma^{\pm } \end{array}\right. \end{array}$$

By testing Equation (1) with $w_{u}\in H^{1}\left(\Omega \setminus \Gamma \right)$ and applying integration by parts in conjunction with Equation (2), we obtain

(3) $$\int_{\Omega \setminus \Gamma }C_{e}:\nabla^{s}u:\nabla^{s}w_{u}\;dV=\int_{\partial \Omega_{t}}t_{N}\cdot w_{u}\;dS+\int_{\Gamma }p_{f}\;\left[\;\left[w_{u}\cdot n_{\Gamma }\right]\;\right]\;dS.$$

where [[·]] denotes a jump quantity over Γ. Given $p_{f}$ and the crack topology Γ, the weak form in Equation (3) can be interpreted as the first-order optimality condition of the total potential energy functional defined in Equation (4).

(4) $$P:=\int_{\Omega \setminus \Gamma }W\;\left(u\right)\;dV-\int_{\partial \Omega_{t}}t_{N}\cdot u\;dS-\int_{\Gamma }p_{f}\;\left[\;\left[u\cdot n_{\Gamma }\right]\;\right]\;dS,$$

with

(5) $$W(u):=\frac{1}{2}C_{e}:\nabla^{s}u:\nabla^{s}u$$

being the elastic strain energy density.

### 2.2. Immersed Fracture Boundary Condition

The presence of discontinuity Γ creates both theoretical and numerical challenges when solving Equation (4). One effective approach to address these issues is to use regularization, i.e., approximating the sharp interface by a diffuse interface, such as the phase-field model [29] as illustrated in Figure 1. However, this diffuse approximation requires reconstructing the pressure that is originally present in a sharp fracture and exhibits a significant gradient perpendicular to the fracture. This process is similar to the immersed boundary method [30], where discontinuities or boundary effects are incorporated into the domain through a smooth transition function.

Accordingly, the last term of Equation (4) can be reformulated as a volume integral over Ω, leading to the regularized energy functional, that is

(6) $$\int_{\Gamma }p_{f}\;\left[\;\left[u(x)\cdot n_{\Gamma }\right]\;\right]\;dS\approx -\int_{\Omega }p_{f}^{\prime }\;u(x)\cdot \nabla d(x)\;dV,$$

where *d* is a continuous-order parameter that regularizes the discontinuous fracture as a diffuse interface. $p_{f}^{\prime }$ denotes a projected pressure defined as a space- and time-dependent field, which is nonzero only within diffuse regions and can be constructed from the pressure at the nearest point on the fracture.

To approximate the fracture energy within the phase-field framework, we adopt the regularized crack functional proposed in [31,32] given by

(7) $$\Gamma_{\ell }(d)=G_{c}\int_{\Omega }\gamma (d,\nabla d)\;d\Omega ,$$

where d∈[0,1] is the phase-field variable, with d=0 corresponding to the undamaged state and d=1 corresponding to the fully broken state. The parameter $G_{c}>0$ denotes the critical energy release rate, i.e., the fracture toughness of the material. The crack surface density function γ(d,∇d) is defined following [31,33] as

(8) $$\gamma \left(d,\nabla d\right)=\frac{1}{4c_{w}}\left(\frac{w(d)}{\ell }+\ell {\left|\nabla d\right|}^{2}\right).$$

where ℓ>0 denotes the regularization length scale governing the width of the diffusive crack zone [33], and w(d) is a degradation function that vanishes in the unbroken state and penalizes the fractured state.

In the phase-field modeling of brittle fracture, the AT1 model is known for producing a compact support profile of the phase-field variable. Unlike the AT2 model, which yields a smooth but infinitely supported profile, the AT1 model leads to a finite-width damage zone, making it particularly suited for simulating crack initiation and fracture localization [34]. In the AT1 model, the degradation function is chosen as

(9) w(d)=d,

and the normalization constant $c_{w}$ is set to

(10) $$c_{w}=\frac{2}{3},$$

thereby ensuring the convergence of the regularized functional to the classical Griffith fracture energy as ℓ→0.

The phase-field representation can be formulated by solving a homogeneous differential equation as shown in [32].

The diffuse approximation of a sharp fracture also introduces a stiffness degradation to the matrix. For the isotropic phase-field model, the degradation function is taken in the form [32]

(11) $$g(d)=\left(1-\kappa \right){\left(1-d\right)}^{2}+\kappa .$$

and then Equation (4) is rewritten as

(12) $$P:=\int_{\Omega }\frac{1}{2}g(d)C_{e}:\nabla^{s}u:\nabla^{s}u\;dV-\int_{\partial \Omega_{t}}t_{N}\cdot u\;dS+\int_{\Omega }p_{f}^{\prime }\;u\cdot \nabla d\;dV,$$

where 0<κ≪1 is a constant for numerical robustness.

The Cauchy stress is then expressed as

(13) $$\sigma =g(d)C_{e}:\nabla^{s}u.$$

### 2.3. Flow Equation

In the hybrid-dimensional framework, the fluid flow within lower-dimensional fractures is described using lubrication theory originally introduced by Reynolds [35]. Following the reduced-dimensional fracture flow formulation in [16], the governing equation is written as

(14) $$\frac{\partial w}{\partial t}+C_{f}w\frac{\partial p_{f}}{\partial t}=\nabla_{s}\cdot \left(K(w)\left(\nabla_{s}p_{f}\right)\right)+q.$$

Here, *w*, $p_{f}$, $C_{f}$, and *q* denote the fracture aperture, fluid pressure, fluid compressibility, and source/sink term, respectively. The hydraulic conductivity K(w) is aperture dependent and follows the local cubic law [36]:

(15) $$K(w)=\frac{w^{3}}{12\mu_{f}}.$$

Since the phase-field method approximates sharp cracks as smeared interfaces, accurately depicting the crack curve or surface remains challenging. This, in turn, impedes the precise calculation of the crack opening. Accurate representations of discrete fracture networks can be achieved using a crack-path reconstruction method, which will be detailed in the next section. In addition, the crack aperture calculation may also require high computational resources. Despite these limitations, the accurate calculation of crack opening is an essential part of fracture simulation. Similar to the motivation of Shahoveisi et al. [37], the crack opening displacement is estimated using an equation derived from the projection of the strain tensor. Compared with displacement-based crack opening calculations, the proposed approach allows the aperture to be evaluated directly from the local strain field without explicitly tracking crack surfaces. In addition, the formulation is relatively simple compared with more complex aperture evaluation methods, which facilitates efficient implementation within the numerical framework. The aperture can be computed as

(16) $$\begin{array}{c} w=h_{e}\varepsilon_{N}, \end{array}$$

where $h_{e}$ is the mesh element size. The normal strain is calculated based on

(17) $$\begin{array}{c} \varepsilon_{N}=\varepsilon :\left(n⊗n\right), \end{array}$$

where n denotes the unit normal vector to the crack, which can be evaluated using the method developed in our previous work [38]. In the present study, only the opening mode relevant to hydraulic fracturing is considered. The sliding mode is not the dominant factor for fracture. Therefore, the aperture perpendicular to the crack path can be used to compute the permeability K(w) based on the cubic law.

### 2.4. Flow Distribution at Multi-Fracture Intersections

In the treatment of fracture networks, intersection points, such as T-junctions and cross-junctions, are usually treated as connecting nodes of control volumes. At these intersections, the total inflow must be appropriately distributed among the outgoing directions to ensure mass conservation and to reflect the heterogeneity of the geometry. Here we apply a simple general flux allocation algorithm for distributing a total inflow $Q_{\mathrm{in}}$ at an intersection into multiple outgoing branches. The method supports both uniform distribution and weighted allocation based on fracture cross-sectional areas. The specific algorithm is described as follows.

Let *N* denote the number of fracture channels at the intersection, and define $A_{i}$ as the cross-sectional area of channel *i*. The outflow flux $F_{i}$ in each direction is then defined as

(18) $$F_{i}=\frac{Q_{i}}{A_{i}}.$$

If geometric heterogeneity is neglected and all outlet channels are assumed to possess equivalent physical properties, a uniform distribution strategy is adopted as

(19) $$Q_{i}=\frac{Q_{\mathrm{in}}}{N}.$$

If the cross-sectional areas of the outgoing channels are unequal, an area-weighted strategy is employed, in which the total flux is distributed proportionally to each outlet as

(20) $$Q_{i}=Q_{\mathrm{in}}\cdot \frac{A_{i}}{\sum_{j=1}^{N}A_{j}}.$$

In fracture networks, the fluid outflow at a node along a given fracture segment is governed by the mass conservation principle together with Darcy’s law. Along the fracture direction, which is typically one-dimensional, Darcy’s law simplifies to

(21) $$Q=u\cdot A=-\frac{k_{f}}{\mu_{f}}\cdot \frac{\Delta p}{\Delta x}\cdot A,$$

where *u* represents the fluid velocity, $k_{f}$ the fracture hydraulic conductivity, $\mu_{f}$ the dynamic viscosity of the fluid, and *p* the pressure field. For each individual node *i*, the total outflow is the sum of flux along all fracture segments connected to this node with its sign depending on the flow direction:

(22) $$Q_{i}^{\mathrm{out}}=\sum_{j\in N(i)}Q_{ij},$$

where N(i) represents the set of neighboring nodes connected to node *i*, and $Q_{ij}$ denotes the flux from node *i* to node *j*, computed with a local discretization scheme (such as the two-point flux approximation (TPFA) [39,40] or the multi-point flux approximation (MPFA) [41,42]).

## 3. Numerical Implementation

This study proposes a hybrid-dimensional framework for staggeredly coupling phase-field fracture in the solid with Reynolds flow in lower-dimensional cracks. It further permits the dynamic reconstruction of two-dimensional discrete fracture networks (DFNs) from phase-field point clouds and accommodates non-conforming discretizations of the solid and fracture domains.

**Figure 2:**
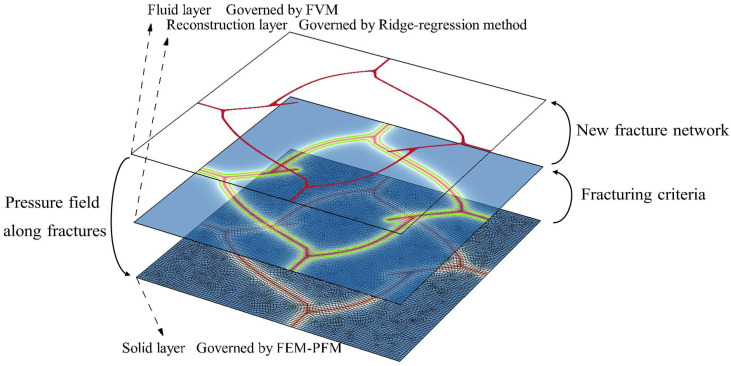
Schematic diagram of the hybrid-dimensional scheme in the coupling process.

The coupling process in planar conditions is described as follows. As demonstrated in Figure 2, the solid domain is discretized by finite element nodes and the fracture domain is regularized using the phase-field method in the bottom solid layer, which is demonstrated with the red–blue contour. The lower-dimensional fluid domain represented by red paths is discretized by the finite volume method in the top fluid layer. The finite element method (FEM) nodes and finite volume method (FVM) nodes use coordinates defined in different-dimensional spaces. Hence the two models are linked based on the crack-path reconstruction method [38]. According to the concept of phase-field point cloud and the optimized ridge-regression method, the discrete fracture networks can be reconstructed dynamically as shown in bright green. The middle reconstruction layer realizes the bidirectional interplay between the hydraulic and mechanical processes.

The problem is spatially discretized using isoparametric elements, while bilinear shape functions are adopted for interpolation according to the standard finite element framework. This interpolation method provides a balance between computational efficiency and numerical accuracy for two-dimensional problems. Therefore, the discrete approximations of u, *d*, and *p* are written as

(23a) $$u=N_{u}\hat{u},\;w_{u}=N_{u}{\hat{w}}_{u},\;\nabla u=B_{u}\hat{u},\;\nabla w_{u}=B_{u}{\hat{w}}_{u}$$

(23b) $$d=N_{d}\hat{d},\;w_{d}=N_{d}{\hat{w}}_{d},\;\nabla d=B_{d}\hat{d},\;\nabla w_{d}=B_{d}{\hat{w}}_{d}$$

(23c) $$p=N_{p}\hat{p},\;w_{p}=N_{p}{\hat{w}}_{p},\;\nabla p=B_{p}\hat{p},\;\nabla w_{p}=B_{p}{\hat{w}}_{p}$$

A staggered scheme is employed to solve the d-(p-u) three-field problem, which is a robust scheme proposed by Miehe et al. [32]. During each time interval $\left[t_{i-1},t_{i}\right]$, it is assumed that all the variables are known at time $t_{i-1}$. The time step size is denoted by Δt. The staggered solution procedure for the three-field coupled problem is outlined in Algorithm 1.
**Algorithm 1** Staggered scheme for the three-field coupling problem.**Input:**  $d_{i-1}$, $p_{i-1}$, $u_{i-1}$ at time step $t_{i-1}$
**Output:**  $d_{i}$, $p_{i}$, $u_{i}$ at time step $t_{i}$
       Initialize the *d*–(p–u) iteration counter k=0       Set $d_{i}^{k}=d_{i-1}$, $p_{i}^{k}=p_{i-1}$, $u_{i}^{k}=u_{i-1}$       **while** $err>tol\;\wedge \;k<k_{\max}$ 
**do**           k←k+1           Solve the phase-field equation to obtain $d_{i}^{k}$ with fixed $p_{i}^{k-1}$ and $u_{i}^{k-1}$           Initialize the *p*–u iteration counter j=0           Set $p_{i}^{k,j}=p_{i}^{k-1}$ and $u_{i}^{k,j}=u_{i}^{k-1}$           **while** $err>tol\;\wedge \;j<j_{\max}$ **do**                j←j+1                Solve the fluid flow equation to obtain $p_{i}^{k,j}$ with fixed $u_{i}^{k,j-1}$ and $d_{i}^{k}$                Solve the momentum equation to obtain $u_{i}^{k,j}$ with fixed $p_{i}^{k,j-1}$ and $d_{i}^{k}$                Compute the error as                $err=\max\;\left(∥p_{i}^{k,j}-p_{i}^{k,j-1}∥,∥u_{i}^{k,j}-u_{i}^{k,j-1}∥\right)$           **end while**           Update $p_{i}^{k}=p_{i}^{k,j}$ and $u_{i}^{k}=u_{i}^{k,j}$           Compute the error as           $err=\max\;\left(∥d_{i}^{k}-d_{i}^{k-1}∥,∥p_{i}^{k}-p_{i}^{k-1}∥,∥u_{i}^{k}-u_{i}^{k-1}∥\right)$       **end while**       Set $d_{i}=d_{i}^{k}$, $p_{i}=p_{i}^{k}$, and $u_{i}=u_{i}^{k}$


### 3.1. The Equilibrium Equation

The weak form of the momentum balance equation is derived by weighting it with the test function $w_{u}$ and integrating over the entire domain. Correspondingly, the weak form yields

(24) $$R_{u}:=\;\int_{\Omega }\nabla w_{u}^{T}\cdot \sigma \;dV-\int_{\Omega }w_{u}^{T}\cdot p\nabla d\;dV-\int_{\partial \Omega }w_{u}^{T}\cdot t\;dS.$$

Then the Galerkin approximation (23a) is substituted into Equation (24). The discrete equation yields

(25) $$R_{u}:=\int_{\Omega }B_{u}^{T}\frac{\partial \sigma }{\partial \varepsilon }B_{u}\hat{u}\;dV\;{\hat{w}}_{u}-\int_{\Omega }N_{u}^{T}\cdot N_{p}\;\hat{p}\cdot B_{d}\hat{d}\;dV\;{\hat{w}}_{u}-\int_{\partial \Omega }N_{u}^{T}\cdot t\;dS\;{\hat{w}}_{u}$$

(26) $$\begin{array}{cc} =0. & \end{array}$$

### 3.2. The Phase-Field Equation

Likewise, the weak form of the phase-field equation is derived by weighting it with the test function $w_{d}$ and integrating over the entire domain. Correspondingly, the weak form yields

(27) $$\begin{array}{cc} R_{d}: & =\int_{\Omega }w_{d}\;\frac{\partial g(d)}{\partial d}\;H\;dV+\int_{\Omega }w_{d}\;p\;u\cdot \nabla \;dV \end{array}$$

(28) $$\begin{array}{cc} \; & \;\;\;\;+\int_{\Omega }w_{d}\;\frac{G_{c}}{4c_{n}}\;\frac{nd^{n-1}}{\ell }\;dV+\int_{\Omega }\frac{G_{c}}{2c_{n}}\;\ell \;\nabla d\cdot \nabla w_{d}\;dV. \end{array}$$

Substitute the Galerkin approximation (23b) into Equation (27). The discrete equation yields

(29) $$\begin{array}{cc} R_{d}: & =\int_{\Omega }N_{d}^{T}\;\frac{\partial g(d)}{\partial d}\;H\;dV\;{\hat{w}}_{d}+\int_{\Omega }B_{d}^{T}\;N_{p}\;\hat{p}\;N_{u}\;\hat{u}\;dV\;{\hat{w}}_{d}\\ \; & +\int_{\Omega }N_{d}^{T}\;\frac{G_{c}}{4c_{n}}\;\frac{nd^{n-1}}{\ell }\;dV\;{\hat{w}}_{d}+\int_{\Omega }\frac{G_{c}}{2c_{n}}\;\ell \;B_{d}^{T}B_{d}\;\hat{d}\;dV\;{\hat{w}}_{d}\\ \; & =0. \end{array}$$

### 3.3. The Flow Equation

First the mass-conserving differential Equation (14) is integrated for arbitrary control volume Ω in the spatial domain, after which the volume integral is converted into a surface integral by the divergence theorem. The faces of each volume are denoted as ∂Ω. Then the semi-discrete equation is written as Equation (30)

(30) $$\int_{\Omega }\frac{\partial w}{\partial t}\;d\Omega +\int_{\Omega }C_{f}w\frac{\partial p_{f}}{\partial t}\;d\Omega -\int_{\partial \Omega }K(w)\nabla_{s}p_{f}\;d\partial \Omega -\int_{\Omega }q\;d\Omega =0.$$

At time step $t_{i}$, application of the Backward Euler scheme for temporal discretization yields

(31) $$\begin{array}{cc} \frac{\partial w}{\partial t} & =\frac{w^{i}-w^{i-1}}{\Delta t}\\ \frac{\partial p_{f}}{\partial t} & =\frac{{p_{f}}^{i}-{p_{f}}^{i-1}}{\Delta t} \end{array}$$

Substituting Equation (31) into Equation (30) gives

(32) $$\int_{\Omega }\frac{w^{i}-w^{i-1}}{\Delta t}\;d\Omega +\int_{\Omega }C_{f}w^{i}\frac{{p_{f}}^{i}-{p_{f}}^{i-1}}{\Delta t}\;d\Omega -\int_{\partial \Omega }K(w)\nabla_{s}p_{f}\;d\partial \Omega -\int_{\Omega }q\;d\Omega =0.$$

Inspired by the numerical implementation in [43], here we introduce the fixed-stress split scheme proposed by [44] to ensure the stability of the flow Equation (32). To this end, *j* is introduced as the coupling iteration index for the u-p system, with the stress fixed at iteration *j*.

(33) $$\begin{array}{c} k_{n}w^{i,j}/h_{e}-p_{f}^{i,j}=k_{n}w^{i,j-1}/h_{e}-p_{f}^{i,j-1}. \end{array}$$

$k_{n}$ can be computed as Equation (34)

(34) $$\begin{array}{c} k_{n}/h_{e}=\frac{\delta p}{\delta w}, \end{array}$$

where $h_{e}$ is the finite element mesh size. δp is a user-defined fluid pressure increment which is much smaller than the real-time pressure in the fracture domain. Then the relevant aperture increment δw can be achieved based on the slight deformation triggered by δp.

For simplicity, $k_{n}/h_{e}$ is denoted as $K_{n}$. Thus, $w^{i,j}$ can be written as

(35) $$w^{i,j}=\frac{p_{f}^{i,j}-p_{f}^{i,j-1}}{K_{n}}+w^{i,j-1}.$$

Substituting Equation (35) into Equation (32) gives

(36) $$\begin{array}{cc} \int_{\Omega }\frac{w^{i,j-1}-w^{i-1}}{\Delta t}\;d\Omega \; & +\int_{\Omega }\frac{p_{f}^{i,j}-p_{f}^{i,j-1}}{K_{n}\Delta t}\;d\Omega +\int_{\Omega }C_{f}w^{i,j-1}\frac{p_{f}^{i,j}-p_{f}^{i-1}}{\Delta t}\;d\Omega \\ & -\int_{\partial \Omega }K(w)\nabla_{s}p_{f}^{i,j}\;d\partial \Omega -\int_{\Omega }q\;d\Omega =0. \end{array}$$

The fracture flow equation is formulated in a reduced spatial dimension. Following the approach adopted in [16,45], the equation is discretized here using the finite volume method. It is solved on a one-dimensional mesh generated with our crack reconstruction method. Given the control volume *k*, the discrete mass-conserving Equation (36) reads

(37) $$\begin{array}{c} \frac{w^{i,j-1}-w^{i-1}}{\Delta t}\delta x+\frac{p_{f}^{i,j}-p_{f}^{i,j-1}}{K_{n}\Delta t}\delta x+C_{f}w^{i,j-1}\frac{p_{f}^{i,j}-p_{f}^{i-1}}{\Delta t}\delta x\\ +{\left[-K(w)\frac{\partial p_{f}}{\partial x}\right]}_{k+1/2}-{\left[-K(w)\frac{\partial p_{f}}{\partial x}\right]}_{k-1/2}-Q^{i}=0, \end{array}$$

where $K(w)=w^{3}/\left(12\mu_{f}\right)$ and the crack aperture *w* is updated using Equation (16) before each *p* solution. *Q* is the integration of the source *q*.

The gradient in the diffusion term of Equation (37) can be further rewritten as

(38) $$\begin{array}{c} {\left(\frac{\partial p_{f}}{\partial x}\right)}_{k+1/2}={\left(\frac{{p_{f}}_{k+1}^{i}-{p_{f}}_{k}^{i}}{\delta x}\right)}_{k+1/2}\\ {\left(\frac{\partial p_{f}}{\partial x}\right)}_{k-1/2}={\left(\frac{{p_{f}}_{k}^{i}-{p_{f}}_{k-1}^{i}}{\delta x}\right)}_{k-1/2} \end{array}$$

The proposed finite volume discretization is implemented using the open-source code JFVM [46], which provides a finite volume framework for solving advection–diffusion equations. Further information and examples are available online.

This section presents the overall coupling procedure of the hybrid-dimensional framework. However, in the subsequent numerical verification section, we assume that the fracture topology (or the corresponding phase-field point cloud) is known in advance. The cloud points can be obtained from micro-seismicity or acoustic emission data in practical engineering and laboratory research [47,48].

## 4. Numerical Verification

In this section, several analytical solutions are employed to validate the proposed fluid–solid coupling scheme. The first three tests assess the hydraulic response of the numerical method, while the fourth tests the mechanical response, that is, the fracture aperture computation in a pre-existing fracture. The final problem assesses the propagation of the hydraulic fracture in the KGD planar model.

### 4.1. A Steady-State Pressure Distribution with Inhomogeneous Permeability

**Figure 3:**
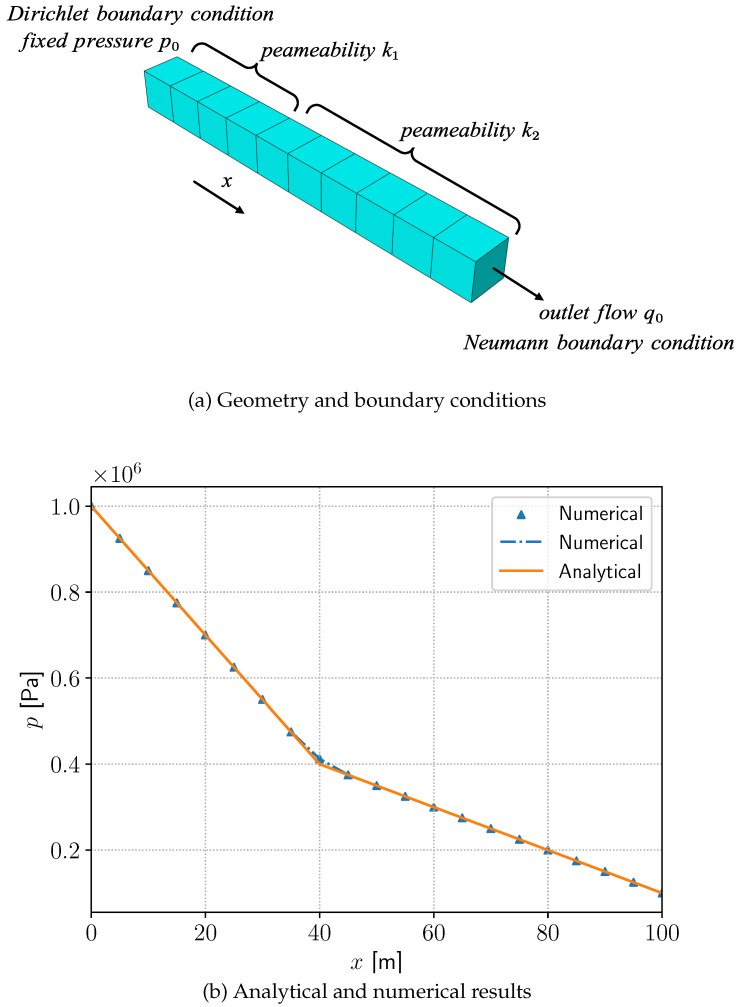
Steady-state pressure distribution with inhomogeneous permeability.

First we verify the viability of the hydraulic module. Since we have captured the lower-dimensional crack paths in our previous work [38] in two dimensions, the one-dimensional fluid flow problem is all we need to focus on. We start with the 1D steady-state pressure distribution with varying permeability in THMC benchmarking [49]. As illustrated in Figure 3a, the computational domain is a beam of length L=100m aligned with the positive *x*-axis and discretized into 20 elements. The three-dimensional model is applied here for demonstration, while the problem is computed in a one-dimensional domain. The crack domain is divided by two kinds of permeabilities $k_{1}=10^{-12}\;m^{2}$ and $k_{2}=3\times 10^{-12}\;m^{2}$ for x<2L/5 and x>2L/5, respectively. The effect of gravity is neglected. The liquid viscosity is μ=1 mPa·s. A Dirichlet boundary condition $p_{0}=1\;\mathrm{MPa}$ is prescribed at x=0m, while a Neumann boundary condition $q=-1.5\times 10^{-5}\;m/s$ is applied at x=Lm. The initial pressure condition is zero for each point on the crack. The analytical solutions to this problem are provided in [49] as

(39) $$\begin{array}{c} p\left(x\right)=\left\{\begin{array}{c} \frac{q\mu }{k_{1}}x+p_{0}\;\;\;\;\;\;\;\;\;\;\;\;\;\;\;\;\;\;\;\;\;\;\;\;\;\;for\;\;x\leq 2L/5\;\\ \frac{q\mu }{k_{2}}x+p_{0}+q\mu \frac{2L}{5}\left(\frac{1}{k_{1}}-\frac{1}{k_{2}}\right)\;\;\;for\;\;x>2L/5 \end{array}\right. \end{array}$$

In this case, the analysis is restricted to the flow field. As shown in Figure 3b, the numerical pressure distribution agrees well with the analytical solution.

### 4.2. A Transient-State Pressure Distribution with Different Boundary Conditions

**Figure 4:**
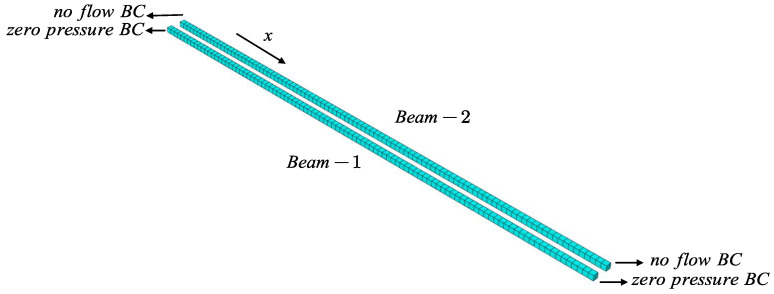
Geometry and boundary conditions of Beam-1 and Beam-2.

Next, we investigate the 1D transient-state pressure distribution with non-zero initial pressure in THMC benchmarking [49]. As shown in Figure 4, there are two beams denoted as Beam-1 and Beam-2 extending along the positive x-axis, reflecting two kinds of boundary conditions. Three-dimensional elements are applied here for demonstration, while the problem is computed in the one-dimensional domain actually. Each beam is 100 m long and divided into 100 elements separately. The whole beam is considered a permeable porous medium filled with liquid of small compressibility. Gravity is neglected. The permeability is assumed to be isotropic with $k=10^{-14}\;m^{2}$, and the fluid viscosity is taken as μ=1.728mPa·s. The matrix porosity ϕ and the fluid compressibility κ are combined as $\phi \kappa =2\times 10^{-10}{\mathrm{Pa}}^{-1}$. The prescribed initial pressure is $p\left(x,t=0\right)=p_{0}\cdot f\left(x\right)$ with $p_{0}=1\mathrm{MPa}$ and f(x) specified below

(40) $$\begin{array}{c} f(x)=\left\{\begin{array}{c} 0\;\;\;\;\;\;\;\;\;\;\;\;\;\;\;\;\;for\;\;\;\;\;0\leq x\leq 0.1L\\ \frac{10}{3L}x-\frac{1}{3}\;\;\;\;\;\;\;for\;\;0.1L\leq x\leq 0.4L\\ 1\;\;\;\;\;\;\;\;\;\;\;\;\;\;\;\;for\;\;0.4L\leq x\leq 0.6L\\ 3-\frac{10}{3L}x\;\;\;\;\;\;\;for\;\;0.6L\leq x\leq 0.9L\\ 0\;\;\;\;\;\;\;\;\;\;\;\;\;\;\;\;for\;\;0.9L\leq x\leq L \end{array}\right. \end{array}$$

The transient pressure field is governed by the fluid diffusion equation derived from the continuity equation and Darcy’s law. The governing equation reads

(41) $$\begin{array}{c} \phi \kappa \frac{\partial p}{\partial t}=\frac{k}{\mu }\nabla \cdot \nabla p+q. \end{array}$$

The zero-pressure boundary condition are prescribed at the ends of Beam-1, while no-flow boundary conditions are imposed at the ends of Beam-2. The analytical solutions of two beams are given in [49,50]. The solutions $p(x,t)/p_{0}$ take the form

(42) $$p_{1}(x,t)=\sum_{n=1}^{\infty }\sin\frac{n\pi x}{L}\exp\left(-\frac{k}{\phi \mu \kappa }n^{2}\pi^{2}\frac{t}{L^{2}}\right)\times \frac{80}{3{\left(n\pi \right)}^{2}}\sin\frac{n\pi }{2}\sin\frac{n\pi }{4}\sin\frac{3n\pi }{20}$$

(43) $$p_{2}\left(x,t\right)=\frac{1}{2}+\sum_{n=1}^{\infty }\cos\frac{n\pi x}{L}\exp\left(-\frac{k}{\phi \mu \kappa }n^{2}\pi^{2}\frac{t}{L^{2}}\right)\times \frac{80}{3{\left(n\pi \right)}^{2}}\cos\frac{n\pi }{2}\sin\frac{n\pi }{4}\sin\frac{3n\pi }{20}$$

**Figure 5:**
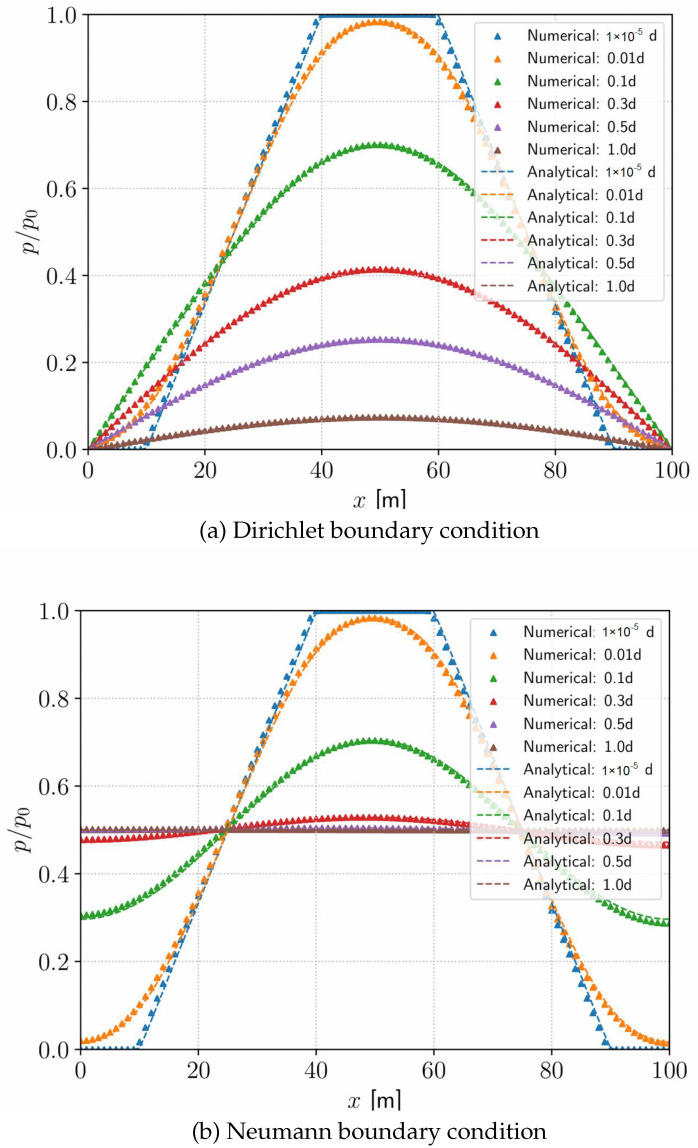
Analytical and numerical results of transient-state pressure distribution under different boundary conditions.

The transient pressure distribution p(x,t) at $t=1\times 10^{-5}$, 0.01, 0.1, 0.3, 0.5 and 1.0 days is shown in Figure 5. When the time step is taken as $1\times 10^{-5}$ days, the fluid pressure distribution is extremely close to the initial pressure specified by Equation (40). As time proceeds, the pressure in Figure 5a is observed to gradually decrease to 0 under the zero-pressure boundary condition, while the pressure in Figure 5b is found to gradually tend to a constant under the no-flow boundary condition. The simulation results are consistent with the physical expectations. Close agreement is observed between the analytical and numerical pressure distributions.

### 4.3. Pressure Distribution in a Single-Cracked Path

**Figure 6:**
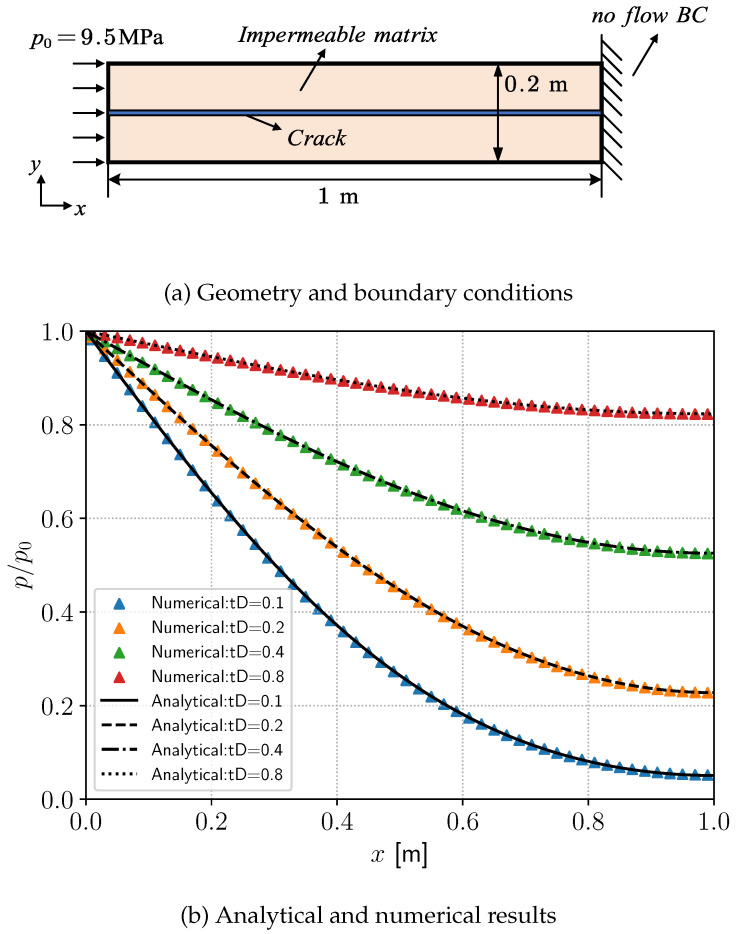
Transient-state pressure distribution in an impermeable rock sample with a single-cracked path.

This section investigates the transient pressure distribution in an impermeable rock specimen containing a single fracture path. You et al. [43] and Song et al. [51] compared the analytical solution of this example with their numerical results. The benchmark parameters adopted in this example are taken from [43]. However, all results are independently computed by the authors using the proposed numerical framework. The geometry and boundary conditions are illustrated in Figure 6a. The left boundary is a Dirichlet condition for $p_{0}=9.5\;$MPa, and the right boundary is a no-flow condition. The permeability is $k=10^{-20\;}m^{2}$ and the fluid viscosity is $\mu =10^{-3\;}\mathrm{Pa}\cdot s$. The fluid compressibility is $C_{f}=4.55\times 10^{-10\;}{\mathrm{Pa}}^{-1}$. By assigning an initial phase-field value d=1 to the fracture domain and using the lower-dimensional fracture reconstruction method, a one-dimensional crack path can be obtained.

The closed form solution of the pressure distribution along the crack [52] is

(44) $$\begin{array}{c} \frac{p(x,t)}{p_{0}}=1+\frac{4}{\pi }\sum_{m=0}^{\infty }\left[\exp\left(-{\left(2m+1\right)}^{2}\left(t_{D}/4\right)\pi^{2}\right)\cos\left(\frac{(2m+1)\pi }{2}\zeta \right)\frac{{\left(-1\right)}^{m+1}}{2m+1}\right], \end{array}$$

where L=1m and $\zeta =\frac{L-x}{L}$.

In this case, $t_{D}$ is defined as

(45) $$\begin{array}{c} t_{D}=\frac{w^{2}t}{12\mu C_{f}L^{2}}, \end{array}$$

where *w* denotes the crack aperture. For clarity, the aperture is assumed to be constant and taken as $w=\sqrt{12\mu C_{f}}$. Figure 6b presents the pressure profiles along the crack at $t_{D}=0.1$, 0.2, 0.4, and 0.8. The numerical results show good agreement with the analytical solution given by Equation (44).

### 4.4. Sneddon Solution for a Pressurized Single Crack

**Figure 7:**
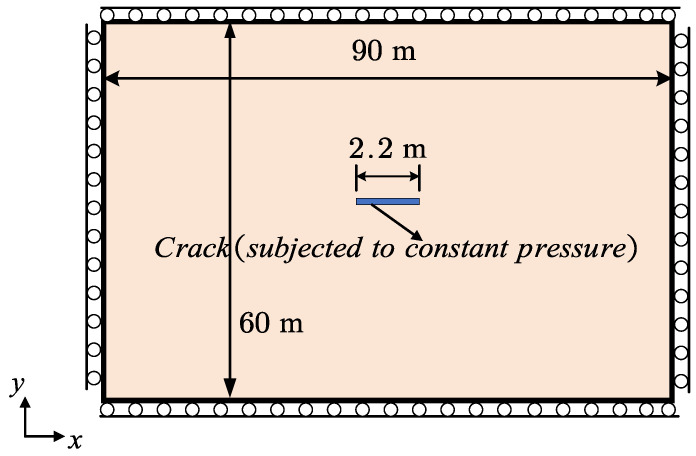
Geometry and boundary conditions of a plate with a single crack under constant internal pressure.

The Sneddon pressurized fracture problem is investigated in this section to verify our crack aperture computation. This problem serves as a classical benchmark for phase-field hydraulic fracture models [16,51,53,54,55,56,57,58]. However, obtaining an accurate crack aperture remains challenging for some models. The geometry and boundary conditions are shown in Figure 7. A pre-existing crack of length 2.2m is subjected to a constant fluid pressure $p_{0}=10^{5}\;\mathrm{Pa}$, which is imposed by setting the phase-field variable to d=1 within the fracture elements.

The analytical vertical displacement along the upper crack surface can be evaluated according to [59]

(46) $$\begin{array}{c} u^{+}\left(x,0\right)=\frac{2p_{0}a_{0}}{E^{\prime }}\sqrt{1-{\left(\frac{x}{a_{0}}\right)}^{2}}, \end{array}$$

where $E^{\prime }=E/\left(1-\nu^{2}\right)$, $a_{0}$ denotes the half-length of the crack, and *x* is the distance measured from the crack center. The material properties are specified as Young’s modulus $E=1.7\times 10^{10}\;\mathrm{Pa}$ and Poisson’s ratio ν=0.2. As discussed in [57], the extra energy near the crack tip should be taken into consideration because of the smeared phase-field fracture. Thus the analytical solution Equation (46) is modified with the effective crack length

(47) $$\begin{array}{c} a_{\mathrm{eff}}=a_{0}\left(1+\frac{\pi \ell_{s}/4}{a_{0}\left(h/4c_{n}\ell_{s}+1\right)}\right). \end{array}$$

**Figure 8:**
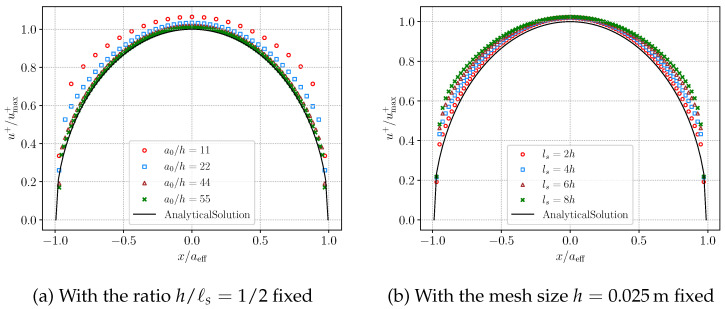
Analytical and numerical crack opening displacements for the AT2 phase-field model with different mesh sizes and length scale parameters.

The effects of the mesh resolution and the length-scale parameter $\ell_{s}$ are then examined. Both the AT1 and AT2 models are considered. The numerical results obtained with the AT2 model are presented in Figure 8. First, the mesh size to length scale ratio is fixed at $h/\ell_{s}=1/2$ as shown in Figure 8a. Four mesh sizes are considered, namely h=0.1m, 0.05m, 0.025m, and 0.02m. In comparison with the analytical solution, the numerical displacement exhibits only a slight sensitivity to mesh refinement. A mesh size of h=0.025m provides sufficient accuracy and yields results consistent with the analytical solution. Next, the mesh size is fixed at h=0.025m to assess the effect of the length scale. The parameter $\ell_{s}$ is taken as 2 h, 4 h, 6 h, and 8 h. As shown in Figure 8b, a smaller length scale leads to numerical results that more closely match the analytical solution.

**Figure 9:**
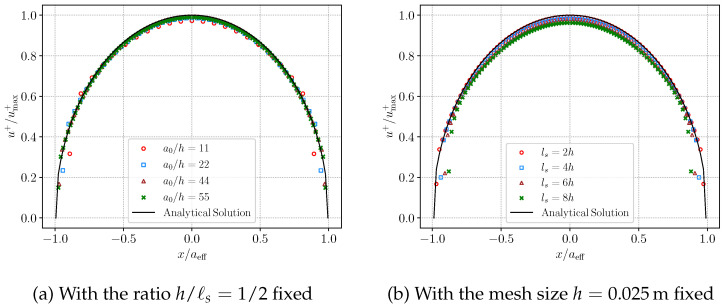
Analytical and numerical crack opening displacements for the AT1 phase-field model with different mesh sizes and length scale parameters.

**Figure 10:**
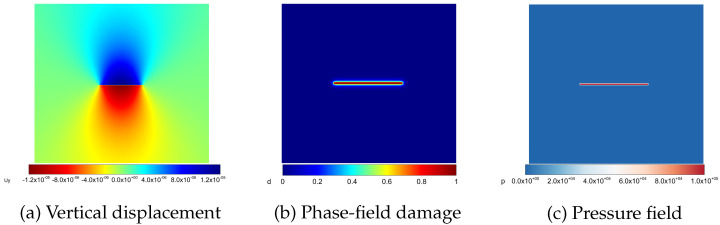
Displacement, phase-field damage, and pressure fields for the Sneddon model.

Next, we turn to the AT1 model shown in Figure 9. A similar investigation is conducted for the AT2 model above. The results indicate that the numerical solutions for the AT1 model are more consistent with the theoretical solution in contrast with the other model. Additionally, the AT1 model is less sensitive to the mesh size. The simulation results are in accordance with the standpoint in [57] that AT1 model computes the crack aperture more accurately. Finally, the simulation profiles of the vertical displacement, phase-field and pressure field for AT1 model with h=0.025m and $\ell_{s}=0.05\;m$ are shown in Figure 10.

#### Comparison with Other Crack Aperture Calculation Method

Although the normal strain-based crack opening displacement (COD) calculation method in Equation (17) has been effectively validated within the Sneddon model, it may not be directly applicable to more complex mesh configurations. In particular, when the element orientation is inconsistent with the crack direction or when unstructured mesh elements are used, the existing COD calculation method requires additional modification to reduce the inaccuracy of the solution due to the element orientation dependence [60].

Therefore, a COD calculation method that is independent of the mesh orientation is required. We introduce a new strain-based method proposed by Fei and Choo [61]:

(48) $$\begin{array}{c} w\approx \frac{(\lambda I+2\mu n⊗n):\varepsilon +p}{\Gamma_{d}\left(d,\nabla d\right)\left(\lambda +2\mu \right)}, \end{array}$$

where λ and μ denote the Lamé constants, I is the identity tensor, and n represents the crack normal vector. Here, ε is the strain tensor, while *p* denotes the pressure.

**Figure 11:**
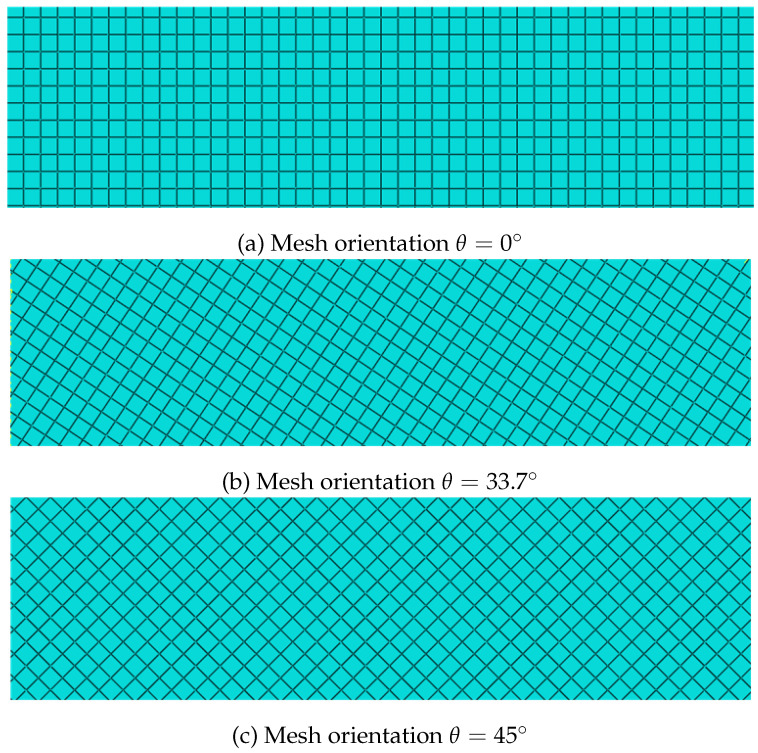
Structured meshes with different orientations for the Sneddon model.

**Figure 12:**
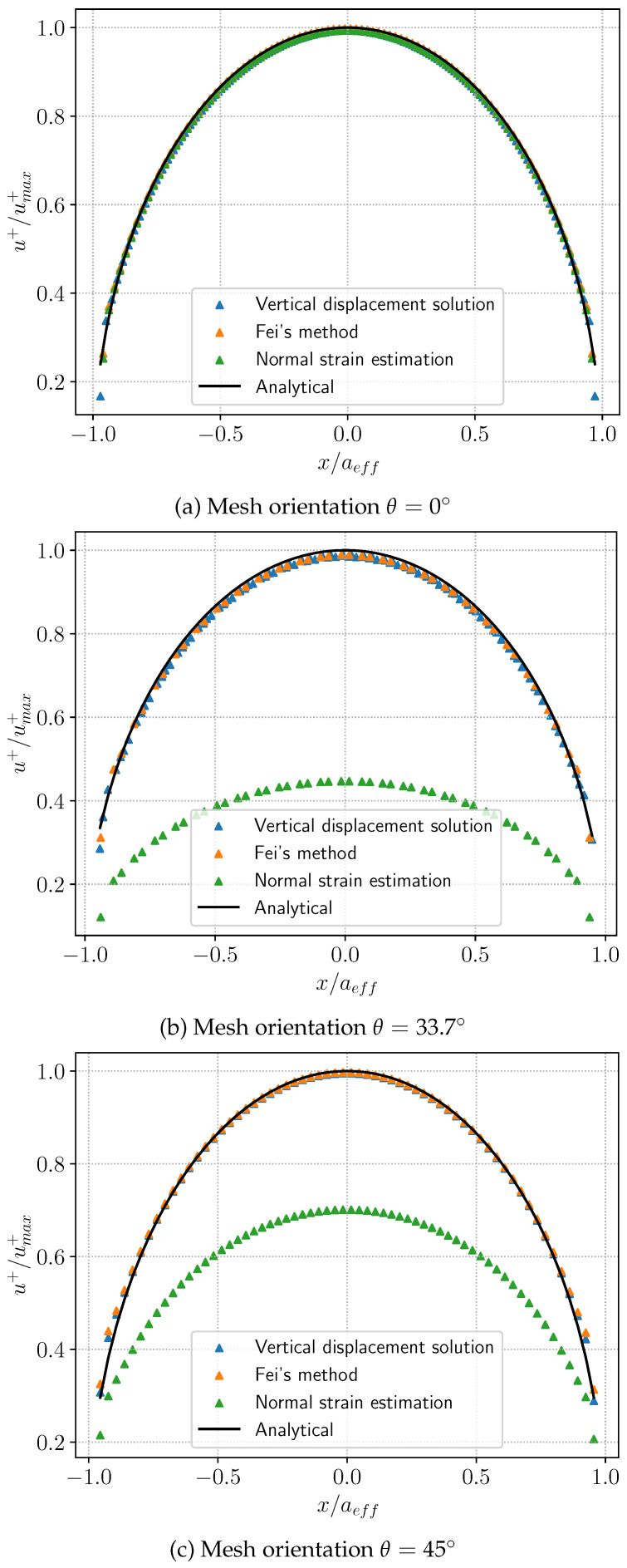
Analytical and numerical results of crack opening displacement (COD) obtained using different aperture calculation methods under various mesh orientations.

We still employ the Sneddon model to evaluate the mesh sensitivity of the COD calculation methods. As illustrated in Figure 11, three types of structured grids are employed for comparison, with angles between the element orientation and the fracture direction set at θ=0°, 33.7°, and 45° respectively. Figure 12 presents a comparative analysis of three COD calculation methods against analytical solutions: (1) the direct calculation using the vertical displacement solution, (2) the aperture solution in Equation (48), and (3) the aperture solution derived from the normal strain in Equation (17). It can be observed that when the orientation of the element aligns with the direction of the fracture, namely the angle θ is 0°, the numerical solutions of the three aforementioned methods exhibit good agreement with the analytical solutions. However, when misalignment occurs, the method based on normal strain demonstrates partial deviation from the analytical solution, whereas Fei’s methodology remains insensitive to element orientation.

This characteristic suggests that Fei’s approach could be integrated with the hybrid-dimensional framework proposed in this study. Such a combination may provide a more reliable aperture estimation for unstructured meshes and complex fracture geometries, which will be investigated in future work.

### 4.5. KGD Model for Hydraulic Fracture Propagation

**Figure 13:**
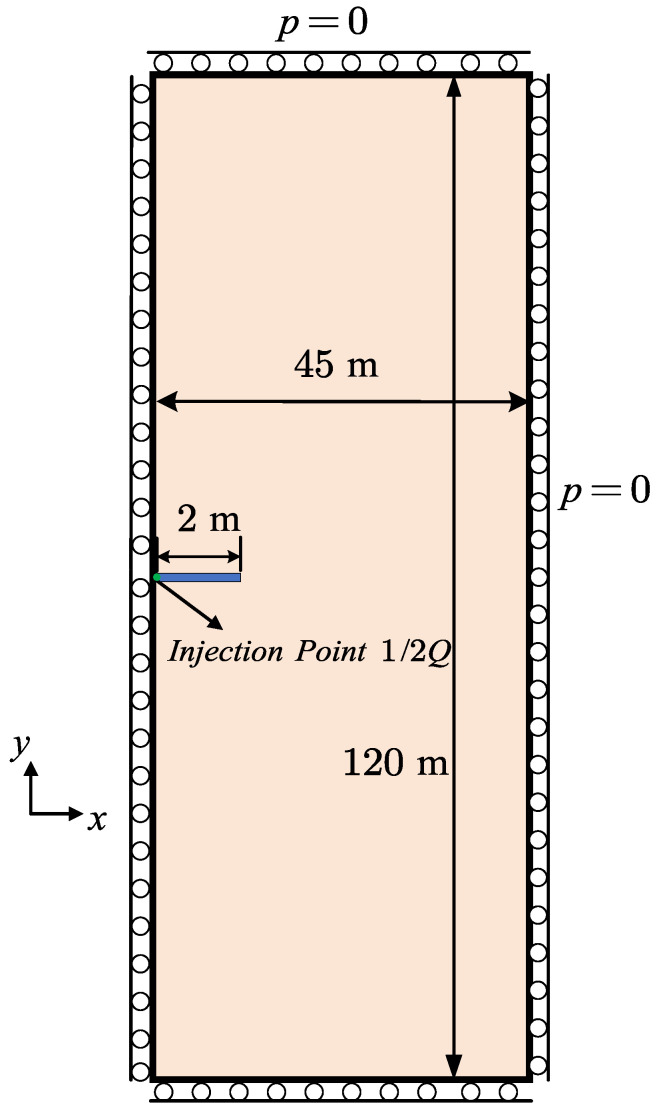
Geometry and boundary conditions of the KGD model.

In the last section, the KGD (Khristianovic–Geertsma–de Klerk) model [62,63] is investigated to verify our hybrid-dimensional iterative coupling scheme for hydraulic fracture in elastic media. The geometry and boundary conditions are demonstrated in Figure 13. For simplicity, a semi-symmetrical structure is taken here. The domain is 120m in height and 45m in width to simulate an infinite plane.

The initial crack is prescribed by setting the phase-field variable to d=1 over a length of 2m. The initial crack aperture is taken as $w_{0}=1\times 10^{-6}\;m$, and the injection rate is prescribed as $Q=2\times 10^{-3}\;m^{2}/s$. The fluid compressibility $C_{f}$ is set to zero to reflect the incompressible fluid assumption adopted in the KGD model. The remaining parameters are listed in Table 1.   

In impermeable elastic media under plane-strain conditions, hydraulic fracture propagation is governed by two dominant dissipation mechanisms: viscous dissipation arising from fluid friction within the fracture and toughness-related dissipation associated with the failure of the solid medium [3]. Garagash [64], Garagash and Detournay [65] give the analytical solution of the toughness-dominated and viscous-dominated regimes respectively. The dimensionless viscosity M, which distinguishes these regimes, is written as

(49) $$M=\frac{K^{\prime }}{{E^{\prime }}^{3/4}{\mu^{\prime }}^{1/4}Q^{1/4}}$$

where *Q* denotes the injection rate of fluid. The other parameters $K^{\prime }$, $E^{\prime }$ and $\mu^{\prime }$ are expressed as

(50) $$K^{\prime }=4\sqrt{\left(\frac{2}{\pi }\right)}K_{\mathrm{IC}},\;K_{\mathrm{IC}}=\sqrt{\frac{EG_{c}}{1-\nu^{2}}},\;E^{\prime }=\frac{E}{1-\nu^{2}},\;\mu^{\prime }=12\mu_{f}$$

The two kinds of regimes are distinguished as follows:

(51) $$\begin{array}{cc} \; & M>4.0\;\;\;\mathrm{toughness}-\mathrm{dominated}\;\mathrm{regime} \end{array}$$

(52) $$\begin{array}{cc} \; & M<1.0\;\;\;\mathrm{viscous}-\mathrm{dominated}\;\mathrm{regime} \end{array}$$

The dimensionless viscosity M with the parameters in this benchmark is calculated as 46.73, indicating a toughness-dominated regime.

#### 4.5.1. Mesh Effect on Numerical Solutions

Now we examine the proposed hybrid-dimensional iterative coupling scheme shown in Figure 2 with varying mesh sizes, types and orientations. Throughout the analysis, the ratio between the crack length scale $\ell_{s}$ and the element size *h* is kept constant at $\ell_{s}/h=2$. In the subsequent simulations, the time step is fixed at Δt=0.1s. During hydraulic fracture propagation, the pressure and crack aperture at the injection point, together with the crack length, are monitored. Both the AT1 and AT2 phase-field models are employed for comparison. It should be noted that, during the initial stage, the numerical curves do not coincide with the analytical solution because the pre-existing crack has not yet propagated, and both the fluid pressure and the fracture aperture increase in an approximately linear manner. Once crack propagation initiates, the numerical results converge and overlap with the analytical solution.

**Figure 14:**
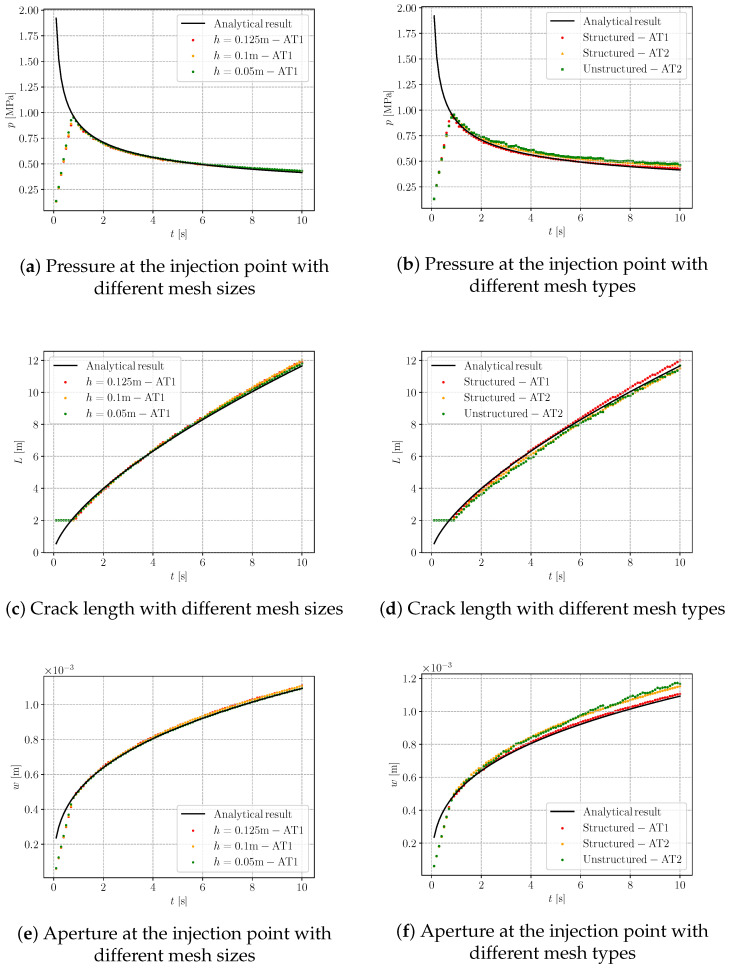
Analytical and numerical results of the KGD model with different mesh sizes and mesh types.

**Figure 15:**
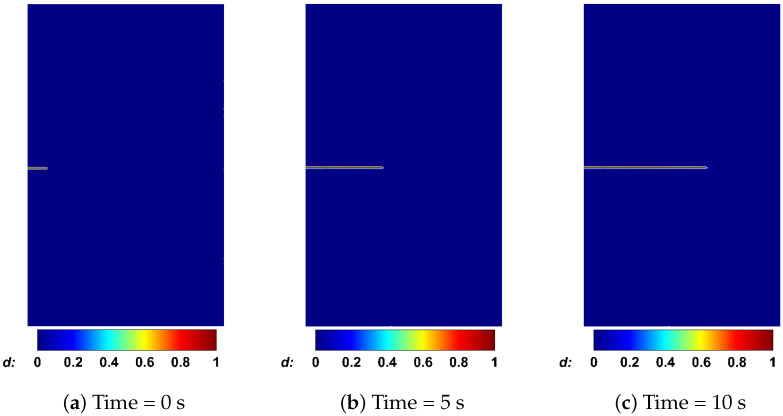
Phase-field profiles at different times with a mesh size of h=0.05m for the KGD model.

We first consider a structured mesh aligned with the horizontal crack propagation direction to examine the influence of mesh size on the numerical results. The AT1 model is employed first, owing to its more accurate prediction of crack opening as demonstrated in the previous section. As shown in Figure 14a,c,e, three mesh sizes, namely h=0.125m, 0.1m, and 0.05m, are considered. All three meshes capture the crack length, crack opening, and fluid pressure evolution with good accuracy. The phase-field distributions at different times for h=0.05m are presented in Figure 15. Although the finest mesh yields results for pressure, crack length, and aperture that are closer to the analytical solutions, the mesh size h=0.1m provides sufficient accuracy while offering higher computational efficiency.

**Figure 16:**
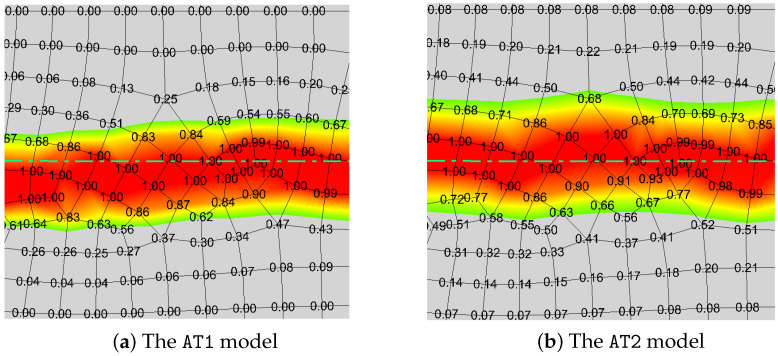
Local *d*-profiles and sampling points for two phase-field models.

Next we turn to the mesh type effect on the simulation results. According to the previous investigation, the mesh size is fixed as h=0.1m for accuracy and efficiency. In the structured mesh, both the AT1 and AT2 recover the general changes of hydraulic fracture in Figure 14b,d,e. However, the AT1 model performs better in calculating the crack aperture in Figure 14e. Additionally, this model is better at simulating crack nucleation [34,66], which is verified in Figure 14d. The fracture initiation is delayed for the AT2 model. Although the AT2 model is not as precise as the former, it shows better robustness in u−p coupling process mentioned in Section 3.3 while dealing with the unstructured mesh. This phenomenon is due to the AT2 model possessing a broader phase-field sampling range, which results in smoother fluid pressure reconstruction combined with the proposed hybrid-dimensional scheme. The comparison of the *d*-profile and sampling range is illustrated in Figure 16. The bright green dash–dot line is marked as the central axis of the standard crack propagation path. The simulated crack path with AT1 deviates from the central axis, while the other remains in the middle position, which has a broader sampling range. Therefore, AT1 is the better choice for structured mesh, while AT2 is more appropriate for unstructured mesh with our reconstruction method.

**Figure 17:**
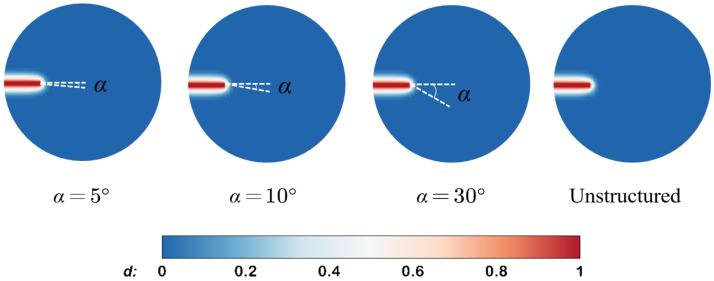
The unaligned mesh with different deflection angle for the KGD model.

**Figure 18:**
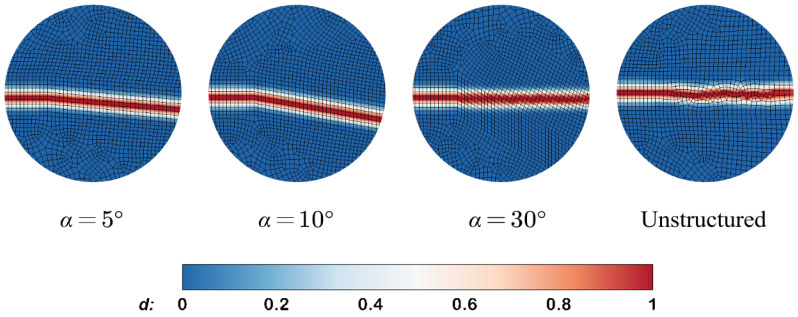
Phase-field distribution near the initial crack tip for different mesh types and orientation.

Finally, the mesh orientation on the hydraulic fracture simulation is investigated. The structured meshes with fixed element size h=0.1m and different deflection angles are considered in Figure 17. An unstructured mesh with identical average size is considered as well. The phase-field profiles around the tip of the initial crack, obtained using different meshes for α=5°,10°,30° and an unstructured mesh, are shown in Figure 18. It can be observed that the phase-field profiles with both AT1 and AT2 models are quite sensitive to the orientation for the first two small deflection angles, α=5° and α=10°. However, the crack propagation results are insensitive to the case of α=30° and to the unstructured mesh. The crack paths remain in the expected medial axis. The simulation results are in accordance with the findings in [67] on the mesh bias sensitivity of phase-field models. Although the investigation argues that the mesh bias can be eliminated using a very fine mesh or a large length scale, the finer mesh comes with additional computational costs. And the larger length scale may affect the accuracy of crack aperture as verified in the former Section 4.4. Hence the standard structured mesh or unstructured mesh can be acceptable considering the computational efficiency and precision, respectively.

The aforementioned comparison of the simulation results and the KGD analytical solution demonstrates that our hybrid-dimensional iterative coupling scheme effectively handles the coupling between the mechanical response of impermeable media and fluid flow in fractures.

#### 4.5.2. Discussion of the KGD Numerical Model

In the following, several aspects of the numerical performance are briefly discussed:

**Iterative stability.** The iterative stability of the coupled KGD model is controlled by the nonlinear interaction between fracture aperture and fluid pressure. In the present benchmark, the iterative process remains overall stable under the adopted time-step size and physical parameters. However, fluctuations may become more likely for larger time steps, higher injection rates, lower material stiffness, or rapid fracture propagation. This stable behavior is mainly attributed to the staggered iterative framework in Algorithm 1 and the fixed-stress algorithm in Section 3.3 used for the flow-mechanics coupling, which together improve the robustness of the coupled solution procedure.
**Convergence of the staggered algorithm.** The staggered strategy solves the displacement, phase-field, and pressure subproblems separately, and exchanges the field variables through outer iterations. The convergence is monitored by the pressure increment norm, displacement increment norm, and phase-field increment norm. The residual tolerance is set to $10^{-10}$, with a maximum of 100 iterations allowed for each time step. Before fracture initiation, the scheme converges rapidly and usually requires fewer than 10 iterations per time step. During fracture propagation, the coupling becomes stronger, and the iteration count increases accordingly. In the strongly coupled stage, the number of iterations may rise to several tens, but each time step can still be completed within 100 iterations.
**Computational cost.** The staggered algorithm has the advantages of simple implementation and strong modularity, but compared with a monolithic scheme, it generally requires more outer iterations. For a relatively simple geometry such as the KGD model, the overall computational cost remains controllable. A major difference from many existing studies is that the flow inside the fracture is solved in a reduced-dimensional form, which greatly lowers the computational cost of the flow problem. For this example, a full-domain flow simulation would require solving a two-dimensional problem with the number of elements on the order of $10^{5}$ to $10^{6}$, namely up to about 500,000 elements without local mesh refinement and still about 50,000 elements even with local refinement. After dimensional reduction, the flow problem becomes one-dimensional with only about 500 elements, i.e., on the order of $10^{2}$. This reduction by approximately two to three orders of magnitude leads to a significant improvement in computational efficiency.
**Sensitivity of the results to discretization parameters.** The numerical results are mainly affected by the mesh size, the phase-field length scale parameter, and the time-step size. The mesh size influences the resolution of the pressure field, displacement field, and phase-field gradient near the fracture. In the present study, local mesh refinement is used to ensure accuracy in the critical region while controlling the overall number of degrees of freedom. As discussed previously, with mesh refinement, key outputs such as the fracture opening, stress peak, and fracture length become closer to the analytical solution, indicating mesh convergence or mesh independence.

The phase-field length scale parameter should be chosen consistently with the mesh size since it controls the width of the diffusive crack zone. Combined with the results in Section 4.4 for the Sneddon model, the numerical results are not very sensitive to this parameter when it is selected in a reasonable range.

The time-step size affects the stability and convergence of the coupled iterations. A large time step may distort the fracture evolution process, while a smaller one usually improves stability and convergence but increases the total computational cost. In the present example, the adopted time-step size provides a satisfactory balance among accuracy, robustness and efficiency.

### 4.6. Fluid Flow in Discrete Fracture Networks

This part demonstrates the capability of the hybrid-dimensional model to simulate fluid flow within a discrete fracture network. This method reconstructs the DFN topology from phase-field point clouds, which then allows fluid flow on the lower-dimensional DFNs to be computed efficiently.

**Figure 19:**
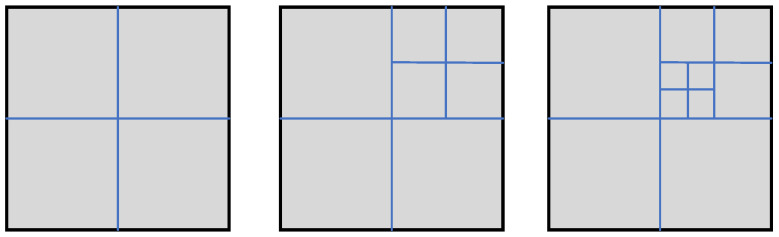
Three idealized 2D grid patterns containing 2, 4, and 6 fractures.

We take a two-dimensional fracture network as an example. Inspired by Geiger et al. [68], three intersecting fracture patterns are shown in Figure 19, containing two, four, and six fractures, respectively, with modified boundary conditions and material properties. The blue region represents high-permeability fracture network channels, while the gray region denotes impermeable matrix blocks. Natural fractures are represented by prescribing the phase-field variable d=1 within the fracture regions, from which the lower-dimensional fracture network is extracted using the reconstruction procedure. The domain size is 2m×2m. A Dirichlet boundary condition of $p_{0}=9.5\;\mathrm{MPa}$ is imposed on the left boundary, while no-flow conditions are prescribed on the remaining boundaries. For simplicity, the fracture permeability is set to $k=10^{-20}\;m^{2}$, and the fluid viscosity is taken as $\mu =10^{-3}\;\mathrm{Pa}\cdot s$. The fluid compressibility is specified as $C_{f}=4.55\times 10^{-10}\;{\mathrm{Pa}}^{-1}$. The flow process is modeled as transient single-phase flow governed by the Reynolds equation, Equation (14). The two-point flux approximation is employed for DFN intersections [39].

**Figure 20:**
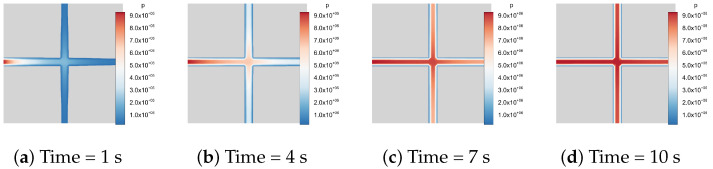
Fluid flow in a 2D fracture pattern containing two fractures.

**Figure 21:**
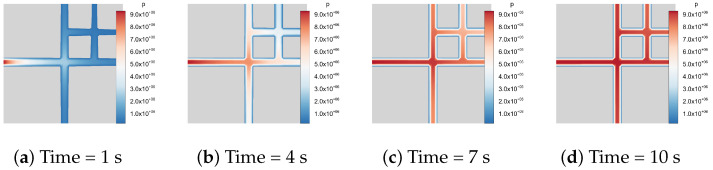
Fluid flow in a 2D fracture pattern containing four fractures.

**Figure 22:**
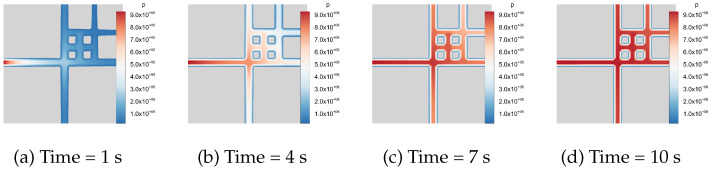
Fluid flow in a 2D fracture pattern containing six fractures.

Figure 20, Figure 21 and Figure 22 illustrate the temporal evolution of the pressure field. The color contours represent the pressure distribution, where blue corresponds to low-pressure regions and red to high-pressure regions. Since the matrix is impermeable, the fluid pressure propagates exclusively through the fracture network from 1s to 10s. At the initial stage, the pressure is mainly localized near the left boundary, after which it gradually spreads throughout the fracture system. Eventually, the flow within the fracture network reaches a stabilized state, and the high-pressure regions become fully established.

**Figure 23:**
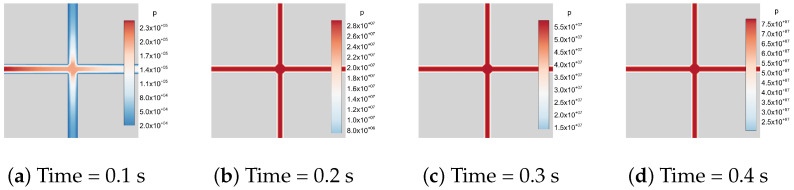
Fluid flow in a 2D fracture pattern containing two fractures under injection boundary conditions.

In addition, a hydro-mechanically coupled case with a constant-rate injection boundary condition is considered. A constant injection rate of $q_{0}=10^{-3}\;m^{2}/s$ is prescribed on the left boundary of the model, whereas no-flow boundary conditions are imposed on the remaining boundaries. The initial fracture aperture is set to $w_{0}=10^{-3}\;m$, and the fluid viscosity is taken as $\mu =10^{-6}\;\mathrm{Pa}\cdot s$. The fluid compressibility is specified as $C_{f}=10^{-10}\;{\mathrm{Pa}}^{-1}$. The flow process is modeled as transient single-phase flow in the toughness-dominated regime. Figure 23 illustrates the temporal evolution of fluid pressure distribution under an injection boundary condition. At the early stage (t=0.1s), the pressure field exhibits a significant spatial gradient along the fractures, with clear evidence of fluid injection from the left boundary. As time progresses, the pressure field becomes more uniformly distributed among the fracture network. A quasi-steady state within the fractures confirms that the system behavior remains in the toughness-dominated regime. Owing to the hybrid-dimensional coupling scheme, the proposed method can be readily extended to address more complex hydro-mechanical interactions between the DFNs and the surrounding solid matrix.

## 5. Discussion

This section provides a brief discussion of the proposed hybrid method, with particular attention to current limitations and possible directions for future research.

In the present study, the formulation is restricted to opening-dominated hydraulic fractures. Since the lower-dimensional flow model is governed by the fracture aperture and the associated cubic-law permeability, only the normal opening component is considered in the present implementation. Shear-induced sliding and mixed-mode fracture effects are not included and remain subjects for future work. At the same time, the current fracture aperture estimation is based on strain projection and is subject to certain limitations related to the mesh orientation and element type. By incorporating the estimation method introduced in Section 4.4, the proposed approach can be extended to more complex fracture geometries and mesh elements.

Another limitation of the present framework lies in the fracture reconstruction algorithm. Although the proposed approach can effectively identify and reconstruct evolving fracture paths in the considered two-dimensional examples, its capability for real-time detection of highly complex and dynamically evolving DFN topologies still requires further improvement, especially in cases with multiple crack branching and merging events. The extension of the current reconstruction strategy to fully three-dimensional fracture systems is also challenging, due to the increased complexity of geometric representation, topological tracking, and coupling with lower-dimensional flow models. In addition, although the reduced-dimensional treatment of fracture flow avoids the direct influence of phase-field diffuseness on the local flow field, the flow response may still be indirectly affected through fracture geometry reconstruction. Therefore, further improvements in reconstruction robustness, computational efficiency, and parallel implementation are needed to extend the method to more realistic large-scale geological problems.

## 6. Conclusions

This paper presents a hybrid-dimensional iterative framework for hydraulic fracturing in deformable geological media with discrete fracture networks. The proposed method combines phase-field based fracture representation, dynamic fracture reconstruction, and lower-dimensional lubrication flow modeling within a staggered coupling scheme. The numerical examples show that the framework can accurately capture fluid pressure, fracture opening, and fracture propagation, while maintaining good numerical performance in terms of stability and convergence. In particular, the benchmark results verify the accuracy of the hydraulic and mechanical solvers, and the DFN examples demonstrate the capability of the method for coupled hydro-mechanical analysis in fractured media. Overall, the proposed framework provides an efficient and flexible approach for simulating fluid-driven fracture processes in complex geological systems. On this basis, we are further developing fluid flow modules to account for different fracture regimes, including toughness-dominated and viscosity-dominated regimes. In future work, improved fracture reconstruction algorithms and iterative coupling frameworks will be developed to address larger-scale and more complex DFN problems.

## Figures and Tables

**Table 1:** Parameters for the KGD model.

Parameter Name	Value	Unit
Young’s modulus (*E*)	$1.7\times 10^{9}$	Pa
Poisson’s ratio (ν)	0.2	-
Critical surface energy release rate ($G_{c}$)	300	N/m
Fluid viscosity ($\mu_{f}$)	$1\times 10^{-8}$	Pa·s
Injection rate (*Q*)	$2\times 10^{-3}$	$m^{2}/s$

## Data Availability

The data supporting the findings of this study are available from the corresponding author upon reasonable request.

## Abbreviations

The following abbreviations are used in this manuscript:

COD	Crack Opening Displacement
DFN	Discrete Fracture Network
FEM	Finite Element Method
FVM	Finite Volume Method
KGD	Khristianovic–Geertsma–de Klerk
MPFA	Multi-Point Flux Approximation
TPFA	Two-Point Flux Approximation
